# Supplementary material for: Impact of Hypoxia on Carbon Ion Therapy in Glioblastoma Cells: Modulation by LET and Hypoxia-Dependent Genes
Source: Cancers (Basel). 2020 Jul 23;12(8):2019. doi: 10.3390/cancers12082019 (PMC7464439; doi:10.3390/cancers12082019)
Supplement: Supplementary file 1 [file cancers-12-02019-s001.pdf]

cancers

MDPI

# Supplementary Materials: Impact of Hypoxia on Carbon Ion Therapy in Glioblastoma Cells: Modulation by LET and Hypoxia-Dependent Genes

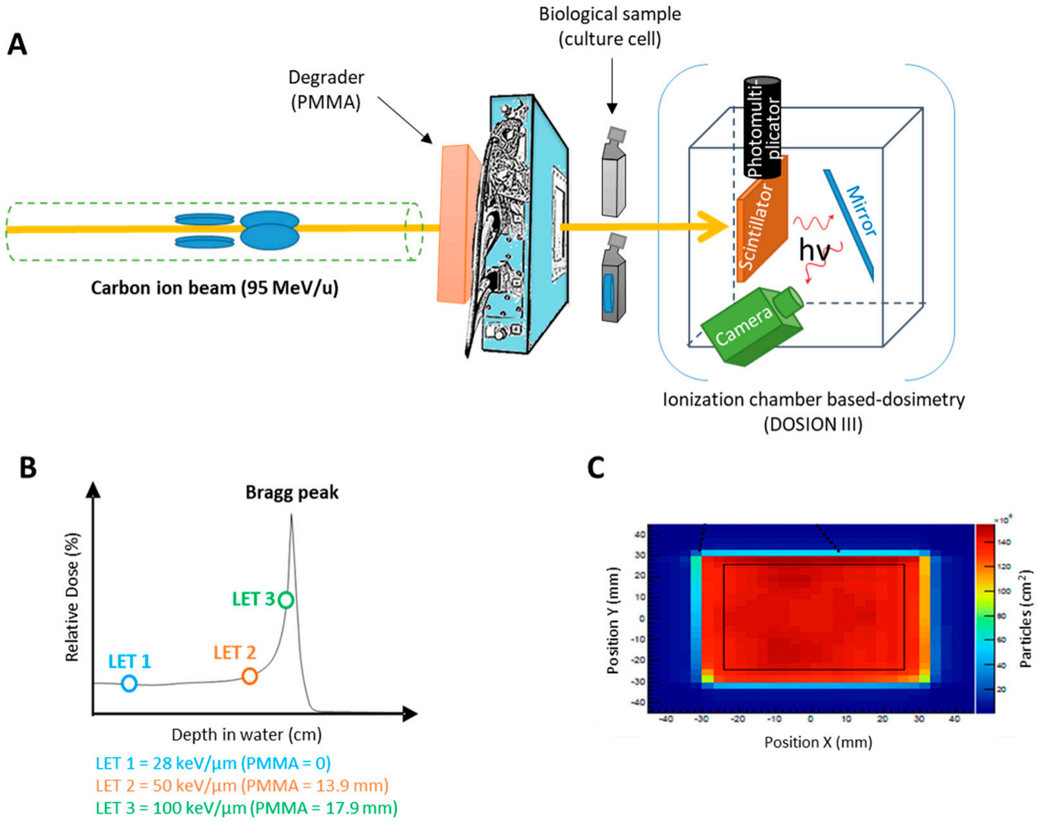


**Figure S1:** Experimental setup for carbon ion irradiation. (**A**) The glioblastoma cells were exposed to carbon ions beam at the energy of 95MeV/u and the delivered doses were checked for each culture flask by ionisation chamber based-dosimetry, (dosimeter DOSION III), placed just behind the culture flasks. (**B**) The LET values were modulated by positioning degrader (several thicknesses of PMMA-poly methyl methacrylate) in front of the culture flasks: 28 keV/µm (PMMA = 0 mm), 50 keV/µm (PMMA = 13.9 mm) and 100 keV/µm (PMMA = 17.9 mm). These LET values were chosen to investigate the glioblastoma response to carbon ion radiotherapy for 3 characteristic points (before, at the beginning and maximum of the monoenergetic Bragg peak). (**C**) Representative dose map of carbon ions estimated from dosimeter measurements on a culture flask. The dose is uniformly deposited and is not variable compared to the dose requested.

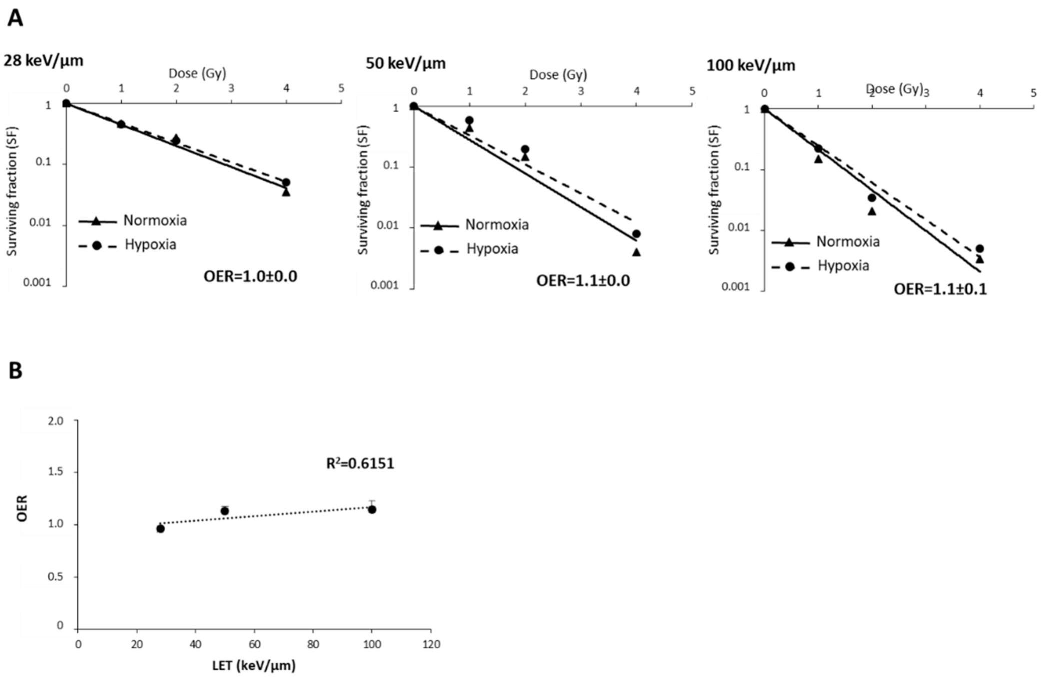


**Figure S2:** The efficacy of carbon ion irradiation on U251 cells is independent of oxygen effect (**A**) Survival curves of U251 cells exposed in normoxia (21% $O_2$) or hypoxia (1% $O_2$) to doses ranging from 0 to 4 Gy at different LET values (28 keV/µm, 50 KeV/µm and 100 keV/µm). For each LET, OER quantification was performed from D37 determined in normoxia and hypoxia. (**B**) Positive correlation between OER and LET values. Mean ± SD, N = 3 different experiments performed in triplicate (n = 3) for normoxia and hypoxia conditions. Non-significant difference observed between normoxia and hypoxia curves whatever the LET value (two-way ANOVA—oxygen and dose effects).

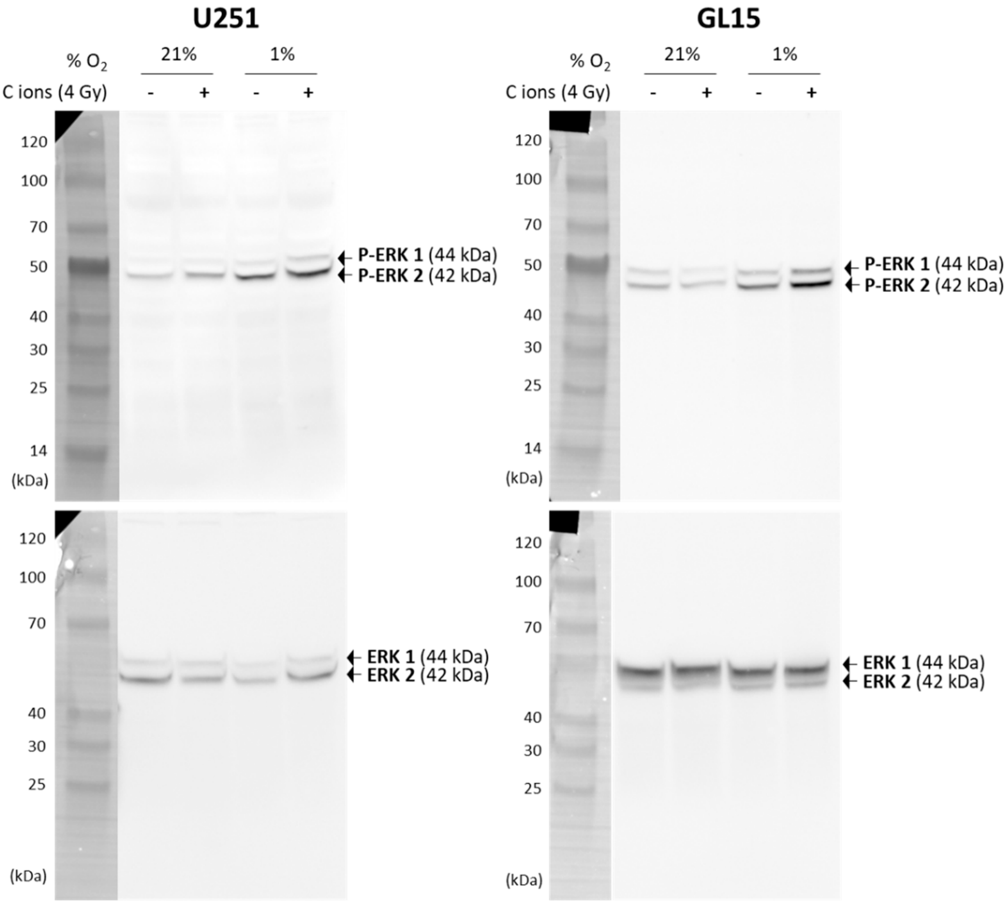


**Figure S3:** Representative whole blots. The whole blots show all the bands for phospho-ERK 1/2 and total ERK 1/2 with all molecular weight markers on the Western performed on protein lysates from U251 and GL15 cells.

© 2019 by the authors. Licensee MDPI, Basel, Switzerland. This article is an open access article distributed under the terms and conditions of the Creative Commons Attribution (CC BY) license (http://creativecommons.org/licenses/by/4.0/).
